# Transferred Patients by Fars Province’s Helicopter Emergency Medical Service (HEMS); A 2-Years Cross-Sectional Study in Southern Iran

**DOI:** 10.30476/BEAT.2021.86919

**Published:** 2021-01

**Authors:** Seyed Mahmoudreza Sajjadi, Fatemeh Rahmanian Koushkaki, Razieh Sadat Mousavi-Roknabadi, Faramarz Farahmand, Afsaneh Dehbozorgi, Hadid Hamrah, Mohammad Javad Moradian

**Affiliations:** 1 *Trauma Research Center, Shahid Rajaee (Emtiaz) Trauma Hospital, Shiraz University of Medical Sciences, Shiraz, Iran *; 2 *Student Research Committee, Department of Emergency Medicine, Faculty of Medicine, Shiraz University of Medical Sciences, Namazee Hospital, Shiraz, Iran*; 3 *Community Medicine Specialist, Department of Emergency Medicine, Faculty of Medicine, Shiraz University of Medical Sciences, Namazee Hospital, Shiraz, Iran*; 4 *Department of Emergency Medicine, Faculty of Medicine, Shiraz University of Medical Sciences, Namazee Hospital, Shiraz, Iran*

**Keywords:** Emergency medical services, Air ambulances, Helicopter ambulance, Trauma

## Abstract

**Objective:**

To investigate the patients transferred by helicopters, as well as an emergent medical services that were performed for them.

**Methods:**

In this retrospective cross-sectional study, all patients who were transferred by Fars province of Helicopter Emergency Medical Services (HEMS) to Shiraz hospitals, southern Iran (March 2017-March 2019) were investigated. Patients’ information was collected and analyzed includes age, gender, dispatch reason, trauma mechanisms, take hold of emergent medical services, as well as the air transportation time, time between dispatch from the origin hospital and starting the procedures, and patients’ outcome.

**Results:**

Eighty-three patients were enrolled with the mean±SD age of 36.9±19.47 years that 75.9% had trauma (*p*<0.0001). Mental status deterioration (25.3%) was the most dispatched indications. The mortality rate was 13.25% totally (11.11% in traumatic vs. 10% in non-traumatic). The mean±SD of air transportation time was significantly lower than ground transportation in both traumatic (*p*=0.0013) and non-traumatic (*p*<0.0001) patients. Also, the mean±SD of time between dispatch from the origin hospital and starting the procedures was statistically lower in air transportation in both traumatic (*p*=0.0028) and non-traumatic (*p*=0.0017) patients.

**Conclusion:**

Most of the patients transferred by HEMS were traumatic. The air transportation time as well as the time between dispatches from the origin hospital to the starting of the procedures were significantly lower in HEMS in comparison with ground transportation for both traumatic and non-traumatic patients.

## Introduction

Helicopter Emergency Medical Services' (HEM) purpose is to provide specialized services for patient’s triage, treatment, and rapid transfer directly to the trauma center for providing definitive treatment [1, 2]. An air ambulance may be the only means of transportation when access the patient is difficult [3].

When deciding about HEMS using, many factors are important include access to higher-level pre-hospital care that HEMS staff provide, faster access to the main trauma center (due to difficulties of geographical access), simultaneous extract of several injured patients need, and access of local communities to ground emergency medical services (GEMS) centers. In many systems, HEMS staff receive Advanced Life Support (ALS) training that may not be available in rural EMS systems [2].

It is proved that HEMS is economical for various clinical conditions if administered carefully. Gearhart *et al*., [4] compared the HEMS with other conventional means of emergency transportations. They reported that it’s an affordable method of transportation in cases which helicopter transmission increases the survival rate. Ringburg *et al*., [5] showed that helicopter transportation is economical. Total planned medical expenses of HEMS in urban and rural areas are 3,308 and 4,962 $, respectively. The cost per travel distance for urban and rural areas are 21.53 and 32.30 $ per mile, respectively.National Trauma Data Bank study conducted by Galvagno et al., calculated that the total costs of HEMS was 325,000 per each saved life in first-level trauma centers [6].

The introduction of HEMS impacts was controversial on the health outcomes of trauma patients [6]. Besides, its pros and cons are not yet known [7]. The results of some studies may be biased due to methodological errors such as the heterogeneity of health care systems (e.g., using physicians or nurses as the HEMS staff) [6]. In-hospital mortality is the most important outcome of the HEMS for multiple trauma patients [6, 8-11]. On the other hand, some studies supported using of HEMS [12, 13].

According to our knowledge, few studies investigated the HEMS in Iran [14-16] and the fact that Shiraz, the capital of Fars province has several hospitals that can host HEMS, the current study's objective was to investigate the patients transferred by helicopters as well as emergent medical services that were performed for them.

## Materials and Methods

Study Design and Population

The current retrospective cross-sectional study (Aug-Dec 2019) was conducted on all patients’ medical records who were transferred by Fars province HEMS to Shiraz hospitals (Namazee, Faghihi, Rajaee, and Hafez), the center of Fars province, southern Iran, from March 2017 to March 2019. Patients who died at the time of arrival to the destination hospitals were excluded.

Study Protocol

The patients’ names and their initial data such as demographic variables (age, gender), the city and the reason of dispatch (traumatic or non-traumatic), the mechanisms of trauma, the indication of dispatch, and the taken pre-hospital and in-hospital emergent medical services less than six hours were collected from Fars province HEMS center. Then, the started time of the procedures as well as the patients’ outcome (discharge or death) were extracted from the patients’ medical files in the destination hospitals and recorded in a data collection form.

To compare the duration of helicopter and ground transportations, the land distance information between the dispatched cities (patients’ locations) and the destination hospitals as well as land estimated times dispatches were obtained from the https://www.google.com/maps website.

A variable was defined: “the estimated time interval of dispatch to the start of the procedure, if the patient was dispatched by ground emergency services” in comparing the air and ground emergency services. To calculate this variable, “air transfer to the hospital duration” was reduced from “total duration of dispatch to start of procedures”. This is the duration of patients’ entrance to the emergency department of the hospital and initiating the procedures. Then, it was summed up by the “ground transfer time of the patient to the destination hospital”. Therefore, if patients were transferred by ground emergency vehicles, the time interval between dispatch and starting of procedures was estimated. It should be noted that 30 minutes was considered for boarding and disembarking patients from the ambulance in ground transportation but this time is hidden for helicopter transportation.

Statistical Analysis

The statistical package for social sciences (SPSS Inc., Chicago, Illinois, USA) version 25 and Medcalc software for Windows were used for statistical analysis through descriptive and analytical tests such as independent t-test, Chi Square, and nonparametric tests. Results are presented as mean±standard deviation (SD) for continues variables and were summarized in number (percentage) for categorical variables. Two-sided *p*-value less than 0.05 and confidence interval (CI) of 95% was considered to be statistically significant.

Ethical Considerations

The current study was supported by Shiraz University of Medical Sciences (grant No. 17434), which was approved by the vice-chancellor of research and technology as well as the local ethics committee (IR.sums.med.rec.1397.344) of Shiraz University of Medical Sciences.

## Results

Totally, as shown in Table 1, 83 patients were enrolled with the mean±SD age of 36.9±19.47 (range, 1-81) years (36.06±19.12 in men vs. 20.43±3.93 in women, *p*=0.550), that 56 (67.5%) of them were men (*p*<0.0001). Sixty-three (75.9%) patients had trauma (*p*<0.0001) which was significantly higher in men (73.02%, *p*<0.0001). The mortality rate was 13.25% totally (11.11% in traumatic vs. 10% in non-traumatic, *p*=0.89). It should be considered that there were no air accidents during the study period and none of the patients died on the way. 

**Table 1:** All patients’ characteristic

**Variables**	**Total** **(n=83)**
Age (year) mean±SD	36.9±19.47
Gender (%)	
Male	56 (67.47)
Female	27 (32.53)
*p*-value	<0.0001*
Status (%)	
Traumatic	63 (75.9)
Non-traumatic	20 (24.1)
*p*-value	<0.0001*
Indication of dispatch	
Mental status deterioration	21 (25.30)
Long bone open fracture	20 (24.10)
Cerebral hemorrhage	16 (19.28)
Injury to the spinal cord	13 (15.66)
Apparent skull fracture	13 (15.66)
Pelvic fracture	10 (12.05)
Acute Coronary Syndrome	9 (10.84)
Falling down	6 (7.23)
Penetrating trauma to the head, neck, chest, abdomen and pelvis	6 (7.23)
Dislocation-fracture with vascular injury	6 (7.23)
Hemothorax	5 (6.02)
Cardiac arrest	5 (6.02)
Pneumothorax	4 (4.82)
Long bone burns ≥2	4 (4.82)
Rib fracture below the nipple line	3 (3.61)
Launch from a motor vehicle	2 (2.41)
Abortion	2 (2.41)
Respiratory arrest	2 (2.41)
Severe trauma <2 or >55 years	1 (1.20)
Hypovolemic shock	1 (1.20)
Cerebral hemorrhage	1 (1.20)
Bleeding in the third trimester	1 (1.20)
Status epilepticus	1 (1.20)
Total	152
Pre-hospital emergent medical services (%)	
Intubation and connecting to ventilator	26 (31.33)
In-hospital emergent medical services (%)	
Blood transfusion	28 (33.73)
Open reduction with internal fixation	18 (21.69)
Central vein insertion	17 (20.48)
Chest tube insertion	8 (9.64)
Lumbar puncture	8 (9.64)
Thoracotomy	7 (8.43)
Fasciotomy	6 (7.23)
Ultrasonography	5 (6.02)
Laparotomy	4 (4.82)
Bifrontal decompressive craniotomy	4 (4.82)
Computed tomography (CT) scan	4 (4.82)
Craniotomy	3 (3.61)
Total	112
City of dispatch (%)	
Sepidan	14 (16.87)
Ghir va Karzin	14 (16.87)
Farashband	11 (13.25)
Firouzabad	9 (10.84)
Beyza	8 (9.64)
Abadeh	6 (7.23)
Eghlid	5 (6.02)
Dasht Arzhan	5 (6.02)
Kazeroun	4 (4.82)
Khaneh Zenyan	3 (3.61)
Kamfirouz	2 (3.41)
Saadat Shahr	1 (1.21)
Unknown	1 (1.21)
Outcome (%)	
Discharged	72 (86.75)
Death	11 (13.25)
*p*-value	<0.0001^a^

^a^ Statistically significant

The mean±SD age of traumatic patients was significantly lower than non-traumatic patients (33.1±16.44 vs. 48.9±23.43, *p*=0.009). Car-car accident (CCA) and car turn over (CTO) were the most mechanisms of trauma (43.9% vs. 39.02%). In general, 130 indications were recorded for dispatch in traumatic patients which mental status deterioration and long bone open fracture were the most of them (33.33% vs. 31.75%). For each patient, at least one emergent medical services were performed (totally 134, 22 pre-hospitals and 112 in-hospital) which blood transfusion was the most of them (44.44%), and 85.71% of the procedures were performed before 202.09 minutes (Table 2).

**Table 2:** Traumatic patients’ characteristics

**Variables**	**Total**
Number (%)	63 (75.9)
Age (year) mean±SD	33.1±16.44
Gender (%)	
Male	46 (73.02)
Female	17 (26.98)
P-value	<0.0001*
Mechanisms of traumatic injury (%)	
Car-car accident (CCA)	18 (28.57)
Car turn over (CTO)	16 (25.40)
Motor-car accident (MCA)	4 (6.35)
Car-person accident (CPA)	2 (3.17)
Motor-person accident (MPA)	1 (1.59)
Total	41
Indication of dispatch (%)	
Mental status deterioration	21 (33.33)
Long bone open fracture	20 (31.75)
Cerebral hemorrhage	16 (25.40)
Injury to the spinal cord	13 (20.63)
Apparent skull fracture	13 (20.63)
Pelvic fracture	10 (15.87)
Falling down	6 (9.52)
Penetrating trauma to the head, neck, chest, abdomen and pelvis	6 (9.52)
Dislocation-fracture with vascular injury	6 (9.52)
Hemothorax	5 (7.94)
Pneumothorax	4 (6.35)
Long bone burns ≥2	4 (6.35)
Rib fracture below the nipple line	3 (4.76)
Launch from a motor vehicle	2 (3.17)
Severe trauma <2 or >55 years	1 (1.59)
Total	130
Pre-hospital emergent medical services (%)	
Intubation and connecting to ventilator	22 (34.92)
In-hospital emergent medical services (%)	
Blood transfusion	28 (44.44)
Open reduction with internal fixation	18 (28.57)
Central vein insertion	17 (26.98)
Chest tube insertion	8 (12.70)
Lumbar puncture	8 (12.70)
Thoracotomy	7 (11.11)
Fasciotomy	6 (9.52)
Ultrasonography	5 (7.94)
Laparotomy	4 (6.35)
Bifrontal decompressive craniotomy	4 (6.35)
Computed tomography (CT) scan	4 (6.35)
Craniotomy	3 (4.76)
Total	112
The number of procedures (%)	
Before 202.09 minutes	54 (85.71)
After 202.09 minutes	9 (14.29)
*p*-value	<0.0001^a^
Outcome (%)	
Discharged	56 (88.89)
Death	7 (11.11)
*p*-value	<0.0001^a^

^a^Statistically significant

The mean±SD of time between dispatch from the origin hospital and starting the procedures was 131.13±77.52 minutes in air transportation, which was compared with estimated time in ground transportation (240.27±73.03 minutes), that this difference was statistically significant (*p*=0.0028) (Table 3).

**Table 3:** Times and distances in transportation of the traumatic patients (n=63)

**City**	**Number (%)**	**Air transportation time (time between the dispatch from the origin hospital and the arrival to the destination hospital) (min)**	**Time between dispatch from the origin hospital and starting the procedure in the destination hospital (min)**	**Ground distance between the origin hospital and the destination hospital (Km)**	**Ground transportation time (estimated time between the origin hospital and the destination hospital in ground transportation) (min)**	**Estimated time between dispatch from the origin hospital and starting the procedure in the destination hospital) (min)**
Sepidan	14 (22.2)	40	58	84	72	90
Ghir va Karzin	12 (19.1)	129	214.5	242	230	322
Firouzabad	8 (12.7)	61	175	124	121	213
Beyza	6 (9.5)	38.5	304.5	46.5	54	146
Farashband	6 (9.5)	35.5	130.8	186	169	261
Abadeh	4 (6.4)	29	102	227	202	294
Dasht Arzhan	4 (6.4)	30	37	65.5	64	156
Eghlid	2 (3.2)	32	92	213	186	278
Kamfirouz	2 (3.2)	49	143	103	97	191
Kazeroun	2 (3.2)	31	65.5	132	128	220
Khaneh Zenyan	2 (3.2)	32	120	45.3	50	142
Unknown	1 (1.6)	ND^a^	ND^a^	ND^a^	ND^a^	ND^a^
Mean±SD^b^	-	46.09±29.13	131.13±77.52	133.48±72.83	124.82±63.73	240.27±73.03

^a^ND=Not determined, ^b^SD=standard deviation

As shown in Table 4, 22 indications were recorded for dispatch in non-traumatic patients which acute coronary syndrome (ACS) and cardiac arrest were the most of them (40.91% vs. 22.73%). For each patient, at least one emergent medical service was performed (totally 23, 4 pre-hospitals, and 19 in-hospital) which coronary angiography was the most of them (45%).

**Table 4:** Non-traumatic patients’ characteristics

**Variables**	**Total**
Number (%)	20 (24.1)
Age (year) mean±SD	48.9±23.43
Gender (%)	
Male	10 (50)
Female	10 (50)
*p*-value	1.00
Indication of dispatch (%)	
Acute Coronary Syndrome	9 (40.91)
Cardiac arrest	5 (22.73)
Abortion	2 (9.09)
Respiratory arrest	2 (9.09)
Hypovolemic shock	1 (4.55)
Cerebral hemorrhage	1 (4.55)
Bleeding in the third trimester	1 (4.55)
Status epilepticus	1 (4.55)
Total	22
Pre-hospital emergent medical services (%)	
Intubation and connecting to ventilator	4 (20)
In-hospital emergent medical services (%)	
Coronary angiography	9 (45)
Ultrasonography	4 (20)
Echocardiography	3 (15)
Adult ICU admission	1 (5)
Blood injection	1 (5)
Heparin Therapy	1 (5)
Total	19
The number of procedures (%)	
Before 239.8 minutes	54 (90)
After 239.8 minutes	9 (10)
*p*-value	<0.0001^a^
Outcome (%)	
Discharged	18 (90)
Death	2 (10)
*p*-value	<0.0001^a^

^a^Statistically significant

The mean±SD of air transportation time in non-traumatic patients was significantly lower than ground transportation (43.70±31.83 vs. 129.40±0.25 minutes, *p*<0.0001). The mean±SD of time between dispatch from the origin hospital and starting the procedures was 131.03±80.60 minutes in air transportation which was statistically higher than the estimated time in ground transportation (251.40±65.03 minutes) (*p*=0.0017) (Table 5).

**Table 5:** Times and distances in transportation of the non-traumatic patients (n=20)

**City**	**Number (%)**	**Air transportation time (time between the dispatch from the origin hospital and the arrival to the destination hospital) (min)**	**Time between dispatch from the origin hospital and starting the procedure in the destination hospital (min)**	**Ground distance between the origin hospital and the destination hospital (Km)**	**Ground transportation time (estimated time between the origin hospital and the destination hospital in ground transportation) (min)**	**Estimated time between ** **dispatch from the origin hospital and starting the procedure in the destination hospital) (min)**
Farashband	5 (25)	35.5	130.8	186	169	261
Eghlid	3 (15)	32	92	213	186	278
Abadeh	2 (10)	29	102	227	202	294
Beyza	2 (10)	38.5	304.5	46.5	54	146
Ghir va Karzin	2 (10)	129	214.5	242	230	322
Kazeroun	2 (10)	31	65.5	132	128	220
Dasht Arzhan	1 (5)	30	37	65.5	64	156
Firouzabad	1 (5)	61	175	124	121	213
Khaneh Zenyan	1 (5)	32	120	45.3	50	142
Saadat Shahr	1 (5)	19	69	118	90	182
Mean±SD^a^	-	43.70±31.83	131.03±80.60	139.93±74.15	129.40±0.25	251.40±65.03

^a^SD=standard deviation

## Discussion

In the current study, all patients transferred by HEMS of Fars province to hospitals of the Shiraz from 2017 to 2019 and the emergent medical services were investigated. As mentioned before, most of the patients were men which is consistent with previous studies [15, 17]. Besides, according to the results, most of the patients who were transferred by HEMS had trauma that most of them were caused by traffic accidents (generally, regardless of the mechanism) followed by worsening of consciousness, long bones open fractures, and cerebral hemorrhage. Taylor *et al*., [18]'s study that was conducted on traumatic patients in Australia, reported that the motor vehicle accidents were the most prevalence followed by motor bike accident and fall from height. Moradian *et al*., [14] reported that the most of the patients used HEMS in Fars province were traumatic. Salimi *et al*., [15] also stated that the most common causes of trauma in patients that were transferred by HEMS in Tehran during 2004 was road traffic accidents and the most common injuries were to the head, face, and limbs.

In the current study, detailed investigation of trauma causes revealed that CCA had the highest frequency followed by CTO and motor-car accident. Andruszkow *et al*., [12] reported that the car accident was the most common type of accidents that resulted in the use of HEMS in Germany followed by fall from height and motor bike accidents. Pedestrian accidents accounted for 4% of HEMS. In this study study, the most common emergent medical services performed for the traumatic patients were blood transfusions, intubation, ventilator connections, placement with internal fixation, and central venous line insertion. Andruszkow *et al*., [13] reported that the most common emergent medical service performed for emergency patients were intravenous fluids injections, sedatives injection, and intubation. In another study [12], they reported sedative injections and intubations as the most common emergent medical services that were performed on the accident scene. In the current study, data about the number of venous fluid injections were not available but given that the most commonly performed procedure was blood transfusion and it can be concluded that almost all patients had fluid therapy.

In the present study, the overall mortality rate was 13.25%, and 11.11% for traumatic patients which was lower than the results reported by Andruszkow *et al*., [12] that reported a mortality rate of 13.8%. Salami *et al*., [15] reported a mortality rate of 11.2% while Champion *et al*., [19] reported a mortality rate of 9.20% in traumatic patients.

Comparison of traumatic patients’ data in the current study with other studies which is used as a reference for evaluating the quality of services provided in trauma, showed that although the mortality rate of traumatic patients dispatched by helicopter was slightly higher in the current study, this difference was not statistically significant.

In the current study, the dispatch’s indications of non-traumatic patients were ACS, cardiac arrest, abortion, and respiratory arrest. In line with the current study, McQueen *et al*., [20] showed that cardiac arrest, chest pain, fainting, and car vehicle accidents (CVA) were the most dispatch’s indications in 628 patients, respectively. Moreover, Kornhall *et al*., [21] reported that chest pain, cardiac arrest, and breath shortness as the main causes of using HEMS in non-traumatic patients.

Some studies compared the efficiency of HEMS and GEMS. For example, Davis *et al*., [22] investigated 10,314 traumatic patients who were transferred by HEMS or GEMS. After controlling the confounding factors, the patient’s odds ratio who were transferred by using the HEMS was high (OR=1.90). According to the findings, HEMS was only useful in patients with a GCS score between 3 and 8 (OR=1.84). Meanwhile, Di Bartolomeo *et al.,* [23] in a prospective cohort study on patients with severe head trauma, found that after controlling the confounding factors, there was no difference in the survival rate of those transferred by HEMS or GEMS.

Schiller *et al*., [24] study on 606 patients with blunt trauma showed that mortality in HEMS was significantly higher than in GEMS. On the other hand, Baxt and Moody reported that the overall mortality rate in HEMS was significantly lower than in GEMS in patients with severe brain injury [25]. In another study on traumatic patients, they found that the survival rate in HEMS was significantly higher than GEMS [26]. Moreover, Andruszkow *et al*., [13] showed that in-hospital survival of almost all trauma patients that used HEMS was better. Also, elderly patients with low energy trauma benefited most from HEMS in comparing with GEMS.

Brown *et al*., [27] found that it resulted in an increased survival rate when HEMS was used to transfer patients with an ISS of at least 15. Thomas *et al*., [28] reported that the mortality rate of patients with blunt trauma who used HEMS was lower than GEMS after adjusting for age, gender, year of referral, ISS, and pre-hospital care. A cost-effectiveness analysis study in Iran showed that there was no statistically significant difference in improving the outcomes of patients that were transferred by helicopter after adjusting for multiple effective factors in patients with moderate to severe brain trauma [16]. According to the results of the current study, air transportation of both traumatic and non-traumatic patients was significantly lower than ground transportation. Besides, the time between dispatches from the origin hospital to the start of the procedures was significantly different in both methods.

One of the strengths of the current study was that Fars province has one of the best equipped and busiest HEMS in the country due to its vastness and mountainous climate. Therefore, investigating the patients who transferred in this province will provided valuable information about characteristics of the patients and performed emergent medical services. On the other hand, the study also had limitations including a low number of patients who were transferred during the study period. Air Emergency Center reported 109 patients but only 83 patients were analyzed due to the incompleteness of data. To obtain comprehensive results, future studies should collect the number of HEMS all around the country, therefore, such services can be used more effectively.

According to the results, most of the patients transferred by HEMS in Fars province were traumatic patients and the main causes of traumatic injuries were CCA, CTO, and motor-car accidents. The overall mortality rate was 13.25% (11.11% for traumatic and 20% for non-traumatic patients). The time between dispatches from the origin hospital to the starting of the procedures was significantly lower in HEMS in comparison with GEMS for both traumatic and non-traumatic patients. Regarding that most of the patients were traumatic, it is necessary to increase the quality of the triage system in HEMS. However, prospective studies with larger sample sizes are necessary.
